# Predictive and prognostic transcriptomic biomarkers in soft tissue sarcomas

**DOI:** 10.1038/s41698-021-00157-4

**Published:** 2021-03-05

**Authors:** Eve Merry, Khin Thway, Robin L. Jones, Paul H. Huang

**Affiliations:** 1 Sarcoma Unit, The Royal Marsden Hospital, London, UK; 2 Division of Molecular Pathology, The Institute of Cancer Research, London, UK; 3 Division of Clinical Studies, The Institute of Cancer Research, London, UK

**Keywords:** Prognostic markers, Sarcoma, Sarcoma

## Abstract

Soft tissue sarcomas (STS) are rare and heterogeneous tumours comprising over 80 different histological subtypes. Treatment options remain limited in advanced STS with high rates of recurrence following resection of localised disease. Prognostication in clinical practice relies predominantly on histological grading systems as well as sarcoma nomograms. Rapid developments in gene expression profiling technologies presented opportunities for applications in sarcoma. Molecular profiling of sarcomas has improved our understanding of the cancer biology of these rare cancers and identified potential novel therapeutic targets. In particular, transcriptomic signatures could play a role in risk classification in sarcoma to aid prognostication. Unlike other solid and haematological malignancies, transcriptomic signatures have not yet reached routine clinical use in sarcomas. Herein, we evaluate early developments in gene expression profiling in sarcomas that laid the foundations for transcriptomic signature development. We discuss the development and clinical evaluation of key transcriptomic biomarker signatures in sarcomas, including Complexity INdex in SARComas (CINSARC), Genomic Grade Index, and hypoxia-associated signatures. Prospective validation of these transcriptomic signatures is required, and prospective trials are in progress to evaluate reliability for clinical application. We anticipate that integration of these gene expression signatures alongside existing prognosticators and other Omics methodologies, including proteomics and DNA methylation analysis, could improve the identification of ‘high-risk’ patients who would benefit from more aggressive or selective treatment strategies. Moving forward, the incorporation of these transcriptomic prognostication signatures in clinical practice will undoubtedly advance precision medicine in the routine clinical management of sarcoma patients.

## Introduction

Soft-tissue sarcomas (STS) are a group of rare, heterogeneous tumours showing variable mesenchymal differentiation, with >80 histological subtypes^1^. At diagnosis, prognostic value is predominantly given to histological grade, tumour size and depth^2–5^. Despite complete surgical resection of localised disease, 40–50% of patients develop locally recurrent or metastatic disease within 5 years^2,6,7^. Treatment options are limited for advanced or metastatic STS^6^. Palliative anthracycline-based chemotherapy has remained first-line therapy for decades, despite low response rates (15–25%)^2,8,9^. Given the propensity of STS to metastasise with significant associated mortality rate, defining tumour prognosis, and new therapeutic targets to enable selective, personalised therapeutic strategies is of considerable interest^2,6^. Molecular biomarkers including transcriptomic signatures and selected genetic markers may provide an avenue for personalised medicine and improved management in these rare diseases. Herein, we provide a comprehensive review of transcriptomic biomarkers currently in development for STS. We conclude by offering a perspective on future avenues for the use of transcriptomic signatures to enhance sarcoma treatment.

### Early developments in gene expression analysis of STS

Histological grading has been used since the late 1970s to predict tumour aggressiveness and prognosis in STS^10^. The most prominent grading systems used are the National Cancer Institute (NCI)^11^ and the French Federation of Cancer Centres Sarcoma Group (FNCLCC)^3^ systems defined in 1984. The latter was shown to be superior in a comparative study within the same population^12^, and this three-grade system is most commonly used in practice^13^. FNCLCC uses three independent histological factors to allocate tumour grade; tumour differentiation, mitotic index, and necrosis^3^. In the majority of STS subtypes, histologic grade is considered the most important prognostic factor, as demonstrated by a multivariate analysis of 1240 patients with localised disease^5^. However, grade is not always a reliable parameter. Limitations of grading systems include the indeterminate prognosis of ‘grade 2’ consisting of approximately 50% of STS assessed with FNCLCC^12^, as well as concerns surrounding reproducibility between independent pathologists, with 75% concordance in FNCLCC grading of STS samples in a study^14^. Finally, grade interpretation from core biopsy samples should be approached with caution since STS are not uniform throughout^15^, and grading systems were developed on whole tumour samples^3,11^.

Introduction of gene expression profiling technologies like DNA microarrays presented an opportunity to develop molecular profiling as a complementary tool to histological grading for improved sarcoma prognostication. Pioneered by Patrick Brown in the late 1990s, DNA microarrays were introduced to probe gene expression alterations at, what was at that time, an unprecedented genomics scale^16^. The ability to accurately measure multiple genes simultaneously ushered an era of molecular tumour analysis with potential for deriving new molecular classification approaches and prognostic tools.

The power of this technology as applied in sarcomas was shown by the Van de Rijn group who reported gene expression profiles of 41 STS with cDNA microarrays^17^. They identified clusters of genes showing specific expression for synovial sarcoma (SS), gastrointestinal stromal tumours (GIST) and two subgroups of leiomyosarcoma (LMS), highlighting subtype-specific gene signatures with potential to identify genes involved in sarcomagenesis. Two studies in the early 2000s used cDNA microarray technology to develop gene expression profiles associated with poor outcome in LMS^18,19^. Lee et al. compared gene expression profiles of 20 primary and 7 metastatic LMS to reveal differential expression of 335 genes, with a subset of 80 ‘discriminating genes’ highly expressed in the metastatic group^19^. Similarly, Ren et al. identified 92 genes that were differentially expressed between low grade, well-differentiated LMS and less well-differentiated, high-grade and metastatic LMS, suggesting that gene expression data could be used to identify clinically aggressive tumours within a specific subtype^18^. Work by Francis et al. identified a 244-gene signature in 89 primary, high-grade STS, which split the cohort into two prognostic subsets^20^. Several hypoxia-related genes, notably *HIF1A* and its targets, alongside genes promoting chromosomal instability, were upregulated in the signature. Although undertaken in small sample sizes and lacking independent validation, these studies provided early promise that gene expression profiling may have prognostic value in sarcomas.

By far the most widespread use of transcriptomics in sarcoma to date has been in molecular classification of distinct sarcoma subtypes. Multiple studies have demonstrated that transcriptomics can readily distinguish between different histological subtypes^17,21–23^. More recently, transcriptomic signatures have shown utility in delineating heterogeneous molecular subgroups within histological subtypes. For instance, Guo et al.^24^, found that LMS is composed of three molecular subtypes with distinct clinical outcomes and biological pathways. A recent study also showed that small round cell sarcomas with distinct fusions (e.g., *CIC*-fused and *BCOR*-rearranged tumours) had unique transcriptomic profiles^25^. Further reinforcing the idea that transcriptomics has a role in the molecular classification of fusion driven sarcomas, the same group showed that consensus clustering of gene expression data in endometrial stromal sarcomas (ESS) was capable of identifying a high grade group comprised of tumours harbouring *BCOR* rearrangements, and a low grade group composed of tumours with a fusion of a *PRC2* zinc finger protein (such as JAZF1 and PHF1)^26^. Interestingly, *YWHAE-NUTM2* positive ESS which is typically considered a clinically aggressive subtype was found to be split in the high grade and low grade clusters, indicative of molecular heterogeneity within this fusion positive ESS^26^.

Taken together, these studies laid the foundation for comprehensive molecular analyses of sarcomas and showed that gene expression profiles may have potential to inform on tumour grade, subtype classification, molecular biology, and relapse risk/prognosis. Our review will focus on the development of prognostic transcriptomic signatures in STS.

### Predictive and prognostic transcriptomic signatures in STS

Advances in RNA sequencing and related profiling technologies and the resulting decrease in the cost of comprehensive transcriptomic analysis has led to the development of several different transcriptomic signatures for STS prognostication. These include Complexity INdex in SARComas (CINSARC), Genomic Grade Index (GGI) and hypoxia-associated signatures (Table 1). We describe the development of these biomarkers, their underlying biological basis and the current status in clinical development.

**Table 1:** Summary of key studies in CINSARC, GGI and hypoxia-based transcriptomic signature development.

Signature	Year	Authors	Title	Journal	Contribution to signature development in sarcomas	References
Complexity INdex in SARComas (CINSARC)	2010	Chibon, F. et al.	Validated prediction of clinical outcome in sarcomas and multiple types of cancer on the basis of a gene expression signature related to genome complexity	*Nat. Med.* **16**, 781–787 (2010).	Development of CINSARC, 67-gene prognostic signature in STS, with validation in STS, GIST, breast carcinoma and lymphoma	^27^
	2012	Lagarde, P. et al.	Mitotic checkpoints and chromosome instability are strong predictors of clinical outcome in gastrointestinal stromal tumours	*Clin. Cancer Res.* **18**, 826–838 (2012).	CINSARC stratification of GIST cohort into high-risk and low-risk (C2 and C1) prognostic groups	^32^
	2013	Lagarde, P. et al.	Chromosome instability accounts for reverse metastatic outcomes of paediatric and adult synovial sarcomas	*J. Clin. Oncol.* **31**, 608–615 (2013).	CINSARC stratification of Synovial sarcoma cohort into high-risk and low-risk (C2 and C1) prognostic groups	^33^
	2013	Italiano, A. et al.	Genetic profiling identifies two classes of soft-tissue leiomyosarcomas with distinct clinical characteristics	*Clin. Cancer Res.* **19**, 1190–1196 (2013).	CINSARC stratification of leiomyosarcoma cohort into high-risk and low-risk (C2 and C1) prognostic groups	^42^
	2020	Croce, S. et al.	The nanocind signature is an independent prognosticator of recurrence and death in uterine leiomyosarcomas	*Clin. Cancer Res.* **26**, 855–861 (2020)	CINSARC stratification of uterine leiomyosarcoma cohort into high-risk and low-risk (C2 and C1) prognostic groups	^41^
Genomic Grade Index (GGI)	2012	Bertucci, F. et al	Genomic Grade Index predicts postoperative clinical outcome of GIST	*Br. J. Cancer* **107**, 1433–1441 (2012)	GGI stratification of GIST cohort into GGI-high and GGI-low prognostic groups	^49^
	2018	Bertucci, F. et al.	The Genomic Grade Index predicts postoperative clinical outcome in patients with soft-tissue sarcoma	*Ann. Oncol.* **29**, 459–465 (2018)	GGI stratification of localised STS cohort into GGI-high and GGI-low prognostic groups	^51^
Hypoxia-based signatures	2016	Aggerholm-Pedersen, N. et al.	A prognostic profile of hypoxia-induced genes for localised high-grade soft tissue sarcoma	*Br. J. Cancer* **115**, 1096–1104 (2016).	Application of a 15-gene hypoxia signature in a cohort of STS to produce high- and low-hypoxia prognostic groups	^62^
	2018	Yang, L. et al.	Validation of a hypoxia-related gene signature in multiple soft tissue sarcoma cohorts	Oncotarget **9**, 3946–3955 (2018).	Development of a STS-specific 24-gene hypoxia signature	^66^

#### Complexity index in sarcomas

The most advanced transcription signature candidate under clinical evaluation is CINSARC. Developed by the group of Frédéric Chibon, CINSARC is a 67-gene signature built from a genomic and transcriptomic analysis of 183 primary non-translocation-related sarcomas^27^. The underlying rationale for characterising these tumours is that non-translocation-related sarcomas, including LMS, undifferentiated pleomorphic sarcomas (UPS) and dedifferentiated liposarcomas, are prone to metastasis^6,7^. A three-step approach was used to define the signature. In the first step, significantly expressed genes were selected according to i) comparative genomic hybridisation (CGH) imbalances ii) FNCLCC grade (specifically grade 3 versus 2) and iii) a previously reported 70-gene chromosome instability signature developed by Carter et al.^28^. In the second step, gene ontology analysis identified pathways associated with histologic grade or genomic imbalance. Finally, in the third step, genes involved in the most overrepresented pathways were selected. These 67 genes comprised i) 37 genes related to CGH imbalance ii) 18 associated with histologic grade and iii) 22 additional genes, not yet included, from the Carter signature. When gene ontology analysis was performed, the 67 genes were found to be related to control of chromosome integrity and mitosis. Specifically, chromosome biogenesis, condensation, alignment and segregation, cell cycle/mitosis and cytokinesis control, and the microtubule-kinesin complex.

The STS training set (*n* = 183), alongside breast cancer and lymphoma samples, was used to generate gene expression centroids for CINSARC grading. There are two CINSARC grades, C1, which is a low CINSARC score comprising ‘good prognosis’ patients, and C2, a high CINSARC score comprising ‘poor prognosis’ patients. In the training cohort, patients in the C1 and C2 groups had 5-year metastasis-free survival (MFS) rates of 75% and 35%, respectively (*p* = 1 × 10^−7^), which was validated in an independent patient cohort (*n* = 127)^27^. While metastasis risk could be predicted by CINSARC (training set: HR 3.7; 95% CI 2.2–6.3 and validation set: HR 2.7; 95% CI 1.02–7.2); FNCLCC grade could only predict metastatic outcome in the validation set and not the training set. Multivariate analysis showed that CINSARC was an independent prognostic factor for metastasis when adjusted for FNCLCC grade, subtype, and vascular/bone involvement. A notable advantage of the CINSARC signature is its ability to split FNCLCC grade 2 into good (C1) or poor (C2) prognosis groups based on metastatic potential. This study was the first to describe a gene expression-based risk classifier, that could potentially identify patients with high metastatic potential. Since its initial development in gene expression microarrays, CINSARC has been extended to other platforms, namely RNA-sequencing and Nanostring probe-based technology in both frozen and formalin-fixed paraffin embedded (FFPE) tissue^29,30^.

CINSARC has also been evaluated in other sarcoma subtypes. GISTs are the most frequent mesenchymal tumours of the gastrointestinal tract, making up 25% of STS^1^. Unlike genomically-complex STS, GISTs are characterised by activating point mutations, most commonly in *KIT* and *PDGFRA*^31^. However, despite optimal surgical resection, 20–40% patients relapse^32^. In the original study, CINSARC categorised a GIST cohort (*n* = 32) into two groups^27^. In C1 no cases developed metastasis, whereas in C2, MFS rates at 5 and 10 years were 61% and 30%, respectively. In a follow-up study, CINSARC stratified 60 primary untreated GISTs into two distinct groups with significantly different MFS^32^. The C1 group (*n* = 32) were metastasis/relapse-free at 5 years, with a 5-year MFS rate of 38% in C2 (*n* = 28).

Synovial sarcoma is characterised by a specific translocation t(X;18), and account for 5–10% of STS^1^. CINSARC has been shown to stratify 58 primary untreated SS into two prognostic groups, C1 and C2, with 5-year MFS rates of 78% and 33%, respectively^33^. This was validated in an independent series of 40 primary untreated SS with similar outcomes. On multivariate analysis, CINSARC was shown to be an independent prognosticator to FNCLCC, and able to split FNCLCC grades 2 and 3 into good and poor prognostic groups in SS. It should be noted that evaluation of CINSARC in translocation-driven sarcomas has been limited to synovial sarcomas and further studies in additional translocation-driven sarcomas is required.

LMS are smooth muscle tumours that represent 10–15% STS^1^. They can occur in almost any location of the body, commonly abdomen, retroperitoneum, large blood vessels, and uterus^34^. Uterine LMS (uLMS) account for 7% of STS and 1–3% of uterine malignancies^34,35^. These are aggressive tumours, with 5-year overall survival (OS) rate of 41% for all International Federation of Gynaecology and Obstetrics (FIGO) stages^36^. FNCLCC grading has failed to predict outcome in uLMS^37^, and WHO could not identify an appropriate grading system for uLMS in 2014^38^. FIGO staging remains the primary prognostic factor, alongside an uLMS-specific nomogram for predicting post-resection 5-year OS^39,40^. There is a need for improved prognosticators to identify patients who might benefit from adjuvant chemotherapy^41^. Italiano et al. showed that CINSARC classified 73 primary LMS, of multiple anatomical locations, into two groups with significantly different MFS^42^. In a retrospective series of 60 uLMS CINSARC divided this cohort, of all FIGO stages, into high-risk (C2) and low-risk (C1) groups^41^. C2 had 5-year relapse-free survival (RFS) rate of 9% compared to 51% in C1. OS rate at 5 years was 29% in C2 and 86% in C1. These findings were validated in an independent series of 32 uLMS from The Cancer Genome Atlas Consortium. FIGO Stage I uLMS are localised, but have a propensity to relapse^43^. CINSARC was able to divide stage I tumours into good and poor prognostic groups, the latter with high risk of relapse and death^41^.

Overall, CINSARC has been shown to be an effective independent risk classifier across a spectrum of subtypes including those harbouring complex karyotypes, point mutations, and translocations^32,33,41,42^.

#### Genomic grade index

Gene expression signatures have reached routine use for risk stratification to aid clinical decision-making in breast cancer^44,45^. Given that there are some similarities in the morphological criteria used to grade both STS and breast cancer [mitotic index in both FNCLCC sarcoma grading and Nottingham (breast cancer) grading systems and tumour differentiation (according to subtype: well, moderately, and poorly in sarcomas; tubule differentiation in breast cancer)], application of breast cancer gene expression tools in STS was of interest^27,46,47^. GGI is a 108-gene mRNA signature developed in a cohort of 64 early-stage oestrogen receptor-positive breast cancers by comparing gene expression profiles of histologic grade 3 and 1 tumours^48^. GGI was shown to reclassify grade 2 breast cancers into two prognostic groups, with high GGI expression (GGI-high) associated with higher risk of recurrence and worse prognosis compared to low GGI expression (GGI-low)^48^.

Bertucci et al. evaluated GGI in 86 non-metastatic resected GISTs^49^. They showed that the GGI-high subgroup had a 5-year RFS rate of 46% (95% CI 28–77, *n* = 20) compared to 91% (95% CI 82–100; *n* = 66) in the GGI-low group (*p* = 1.4 × 10^−6^)^49^. GGI also independently predicted RFS in an independent series of GIST samples (*n* = 60). At present GIST prognostication relies on the Armed Forces Institute of Pathology (AFIP) classification based on tumour size, site, and mitotic rate^50^. GGI-high samples were more frequently associated with poor prognosis variables, including AFIP high-risk tumours^49^. The prognostic performance of GGI was compared to AFIP and both had independent prognostic value on multivariate analysis for predicting relapse risk. GGI was able to further define the intermediate/high-risk AFIP samples into two groups; high-risk and low-risk, with a 5-year RFS rate of 35% (95% CI 17–70) and 73% (95% CI 52–100), respectively (*p* = 8.5 × 10^−3^). To explore the relationship between GGI classification and imatinib response, the authors analysed a small cohort of pre-treatment GISTs (*n* = 28) from patients with advanced primary or recurrent operable GIST treated with 8–12 week neoadjuvant imatinib in a phase II trial (RTOG0132). There was greater tumour shrinkage (evaluated on CT with Response Evaluation Criteria in Solid Tumours [RECIST]) in GGI-high tumours than GGI-low, suggesting that GGI-high tumours were more sensitive to imatinib, with need for larger prospective trials to confirm this.

The same group set out to identify correlations between GGI-based classification and clinicopathological variables in a cohort of localised STS (*n* = 678)^51^. The most frequent histological subtypes were liposarcoma (38%), UPS (30%), and LMS (38%), thus 433 (65%) were defined as ‘genomically complex’. 56% were FNCLCC grade 3, and 41% were classified as GGI-low with 59% GGI-high. The GGI-high subgroup had a poor prognosis with 5-year MFS rate of 53% (95% CI 47–59) compared to 78% (95% CI 72–85) in the GGI-low group (*p* = 3.02 × 10^−11^). Additionally, the GGI-high group was associated with STS in the extremities versus the trunk, complex genetic profiles and FNCLCC grade 3 tumours. GGI was able to stratify STS patients with histologic grades 1 and 2 into two prognostic groups, GGI-high and GGI-low, with different 5-year MFS rates; 59% (95% CI 46–76) and 74% (95% CI 62–87), respectively.

For comparison, CINSARC was applied to the Bertucci et al. GIST cohort (*n* = 86) and C2 and C1 groups defined with a 5-year RFS rate of 67% (95% CI 53–86; *n* = 38) and 92% (95% CI 84–100; *n* = 48), respectively (*p* = 0.01)^49^. Ontology analysis showed that, similar to the C2 subgroup, overexpressed genes in the GGI-high subgroup included those associated with cell cycle control and genome stability. When comparing GGI and CINSARC, there were 39 genes in common^51^. Additionally, strong correlation was observed between GGI and CINSARC classes in a further study by Bertucci et al. of 678 STS, with 71% concordance in allocation to low-risk and high-risk groups^51^. Similar to CINSARC, the GGI signature is composed of genes involved in cell cycle regulation with further work required to determine regulators of GGI gene expression and scope for applications in targeted therapy.

#### Hypoxia-based signatures

Intratumoural hypoxia is considered an adverse prognostic factor for metastatic spread in multiple malignancies^52–55^. An association between tumour hypoxia levels and poor outcome has been observed in STS^54,56–58^. Nordsmark et al. evaluated 28 STS tumours and stratified cases according to tumour oxygenation; tumours with median pO_2_ > 19 mmHg were classified as well-oxygenated and ≤19 mmHg as hypoxic. The hypoxic group had significantly lower disease-specific survival of 40% compared to 77% for well-oxygenated tumours (*p* = 0.05)^54^. Hypoxic tumours also had significantly poorer 5-year OS probability of 28% versus 77% (*p* = 0.01). Another small study of primary, high-grade STS (*n* = 30), treated with neoadjuvant radiotherapy and hyperthermia found that more hypoxic tumours, defined as pO2 median value <10 mmHg pre-treatment, had disease-free survival (DFS) rate of 35% compared to 70% in tumours with median pO2 > 10 mmHg pre-treatment^56^. In addition, metastatic recurrence in eight patients was associated with significantly lower median pre-treatment pO2 compared to those that did not metastasis. Work to identify the hypoxia biomarkers followed; Forker et al. utilised STS specimens from the phase III adjuvant radiotherapy VorteX trial, which assessed whether reduced adjuvant radiotherapy volume could improve limb function in adults with extremity STS without compromising local control^59^. Immunohistochemistry for hypoxia protein marker expression, specifically HIF-1alpha, CAIX, and GLUT1, was performed on tissue microarrays from histologically heterogeneous STS specimens (*n* = 203). CAIX was a significant prognostic biomarker, with worse DFS at 5 years in tumours with >10% CAIX staining compared to ≤10% staining (HR 1.75; 95% CI 1.04–2.94; *p* = 0.037). However, there was concern that there was limited overlap expression of these three hypoxia markers across tumour samples, with co-expression absent in hypoxic samples. This may be a result of significant molecular heterogeneity of sarcoma limiting use of single biomarkers. Robust interpretation of whole tumour hypoxia may be best achieved by assessing multiple-gene response with hypoxia gene expression signatures^59,60^.

Aggerholm-Pedersen et al. applied a 15-gene hypoxia-induced signature, previously developed for head and neck cancer^61^, to stratify STS patients into ‘more hypoxic’ (high hypoxia gene expression) and ‘less hypoxic’ groups in FFPE tissue samples^62^. Fifteen genes were selected from a panel of 30 validated hypoxia-responsive, pH-independent genes, including *LOX, P4HA1, and P4HA2* involved in extracellular matrix modulation, and genes involved in glycolysis such as *SLC2A1, PFKB3*, and *PDK1;* both biological processes influenced by hypoxia^61,63–65^. The signature was used in a test (*n* = 55) and validation cohort (*n* = 77), each made up predominantly of liposarcomas and UPS^62^. In the test cohort, HR for disease-specific mortality was 4.09 (95% CI 1.34–12.46; *p* = 0.013) for ‘more hypoxic’ tumours compared with ‘low hypoxic’ tumours. Recurrent disease was also higher in the ‘more hypoxic’ group compared with the ‘less hypoxic’ group (odds ratio 3.96; 95% CI 0.98–14.7; *p* = 0.03). Similar outcomes were observed in the validation cohort. This study suggests that hypoxia gene signatures could be used as prognostic biomarkers for STS, but was limited by wide confidence intervals in the survival analysis and lower hazard ratio in the validation cohort, requiring further validation in larger cohorts^62^. Furthermore, 16 samples, with corresponding oxygen tension measurements available, were analysed with an unexpected finding: cases with low hypoxia levels (high pO2) were associated with high hypoxic gene expression. Therefore, the study could not conclude that expression of these 15 genes in STS is hypoxia driven.

In another study, Yang et al. developed a STS-specific 24-gene hypoxia signature^66^. To define this signature they first identified 33 genes induced by hypoxia in seven cell lines representative of common STS subtypes in adults. These 33 seed genes were used to derive a hypoxia signature in clinical specimens; the training cohort (*n* = 182) was separated into two groups; high-hypoxia and low-hypoxia, based on unsupervised clustering of the 33 genes, with 24/33 seed genes (73%) significantly upregulated in the high-hypoxia group. Gene set enrichment analysis was undertaken of the entire transcriptomic data for cases in the high-hypoxia group revealing upregulation of 16 hypoxia pathways. Survival analysis showed that ‘high-hypoxia’ tumours had worse 5-year distant metastatic-free survival (DMFS) rate than ‘low-hypoxia’ tumours (HR 2.43; 95% CI 1.49–3.96; *p* = 0.00036), which was validated in two independent cohorts of heterogeneous STS subtypes. The hypoxic signature retained its prognostic significance in multivariable analysis adjusted for histological diagnosis, tumour site, site, gender, and age. However, tumour grade was omitted from the multivariable analysis. Given histologic grade is considered the most reliable prognostic factor for the majority of STS, this is a major study limitation^5^. The 24-gene signature was deemed a superior prognosticator to the earlier 15-gene signature^62^, since the latter only achieved prognostic significance for DMFS on multivariable analysis in 2 of the 3 STS cohorts used in Yang et al’s study^66^. Notably there was overlap of 8 common genes between the signatures.

It has been hypothesised that tumour hypoxia drives genomic instability, and thus promotes tumour aggressiveness and distant spread^67^. Interestingly in Yang et al. study, CINSARC was used as a measure of genome instability, and in a combined analysis of training and validation cohorts, more C2 tumours were found in the high-hypoxia than the low-hypoxia group (76 and 48%, respectively)^66^. Prognostic value of the hypoxic gene signature was enhanced by CINSARC with significantly worse DMFS in combined high-hypoxia/C2 patients than low-hypoxia/C1 tumours (HR 6.74, 95% CI 3.84–11.84, *p* = 3.13 × 10^−11^). This highlights the potential of integrating different transcriptomic biomarker signatures to derive better risk classifiers for sarcoma prognostication.

### Clinical evaluation of transcriptomic biomarkers in STS

Having described transcriptomic biomarker development in STS, here we consider their clinical relevance. Thus far, all published studies have been carried out retrospectively in heterogeneous STS populations. There is a need for prospective clinical trials, ideally randomised, to evaluate these transcriptomic signatures in STS to achieve the necessary level of evidence (LoE) for incorporation into international guidelines and use in the clinical setting^68^.

Transcriptomic signatures could contribute to the much-debated topic of peri-operative chemotherapy in STS. Adjuvant chemotherapy following ‘gold-standard’ surgical resection of localised STS remains a very controversial topic^69–71^. Since the 1980s, a number of adjuvant chemotherapy trials have been published, but most are limited by the inclusion of heterogeneous subtypes, small numbers and suboptimal schedules. In the neoadjuvant setting, a multicentre phase III randomised trial assigned patients (*n* = 328) to 3 or 5 cycles of combined epirubicin/ifosfamide neoadjuvant chemotherapy, concluding that prolonged chemotherapy did not benefit OS^72^. Another phase III randomised trial of 287 patients with localised, high risk (grade 3, size ≥ 5 cm) STS of five histologic subtypes (LMS, high-grade myxoid LPS, SS, MPNST, UPS) found that histology-tailored neoadjuvant chemotherapy was not associated with better DFS or OS compared to standard anthracycline-ifosfamide chemotherapy^73^. Therefore, current international guidelines advise neoadjuvant chemotherapy can be considered in ‘high-risk’ STS^2^. Transcriptomic signatures may have utility in further defining ‘high-risk’ STS patients that might benefit from peri-operative chemotherapy.

At present, of the signatures described, only CINSARC is being evaluated in prospective clinical trials (Table 2). The first is a phase III randomised trial (NCT03805022) assessing whether more intensive peri-operative chemotherapy improves outcome of patients with resectable STS and high-risk CINSARC signature (C2). Control arm A will assess C2 patients treated with three cycles neoadjuvant chemotherapy (doxorubicin/ifosfamide) followed by surgery +/− radiotherapy. In experimental arm B, C2 patients will receive an additional three cycles of chemotherapy, followed by surgery +/− radiotherapy, with a 3rd prospective arm for low-risk CINSARC patients (C1) receiving treatment at the discretion of the investigator. The primary endpoint is metastatic progression-free survival after 3 years of follow-up. The second is a prospective single-arm observational study (NCT02789384) in non-metastatic STS, that aims to validate the prognostic value of CINSARC and correlation with chemotherapy efficacy. Patients will be classified with CINSARC prior to neo-adjuvant anthracycline-based chemotherapy. Following chemotherapy, patients will proceed to surgery +/− radiotherapy, with follow-up for the duration of treatment. The primary outcome of chemotherapy efficacy is RECIST v1.1 response, with further analysis to determine association of CINSARC grading with response and survival.

**Table 2:** CINSARC prospective clinical trials.

Trial number	Phase	Title	Allocation	Intervention model	Primary outcomes	Secondary outcomes
NCT03805022	III	Benefit of intensified peri-operative chemotherapy within High-risk CINSARC patients with resectable soft-tissue sarcomas (CIRSARC)	Randomised	Parallel assignment	- Metastasis progression-free survival [3 years]	- Loco-regional relapse-free survival [3 years] - Progression-free survival [3 years] - Overall survival [3 years] - Best overall response as per RECIST v1.1 [6 months] - Histological response [6 months] - Adverse events/Toxicity as per NCI-CTCAE v5.0 [6 months]
NCT02789384	N/A	Prognostic value of the CINSARC signature and correlation with chemotherapy efficacy in soft-tissue sarcomas. A Biomarker Study. (NEOSarcomics)	N/A	Observational- Single Group Assignment	- Best overall response as per RECIST v1.1 [6 months]	- Histological response [6 months] - Metastasis-free survival [3 years] - Overall survival [3 years] - Adverse events/Toxicity as per NCI-CTCAE v4.0
NCT04307277	III	Interest of peri-operative chemotherapy in patients with CINSARC high-risk localised Grade 1 or 2 soft tissue sarcoma	Randomised	Parallel assignment	- Metastasis-free survival [5 years]	- Disease-free survival [5 years] - Toxicity as per NCI-CTCAE v5.0 - Overall survival [5 years]

Another phase III multicentre randomised trial (NCT04307277) will evaluate peri-operative chemotherapy in C2 patients with resectable FNCLCC grade 1/2 STS. Control arm C2 patients will be randomised to surgical excision +/− radiotherapy, whilst experimental arm C2 patients will receive four cycles of peri-operative anthracycline-based chemotherapy in addition to standard management. A third prospective cohort will include C1 patients treated at the discretion of the investigators. The primary endpoint is MFS. This trial aims to determine whether CINSARC can identify chemo-sensitive tumours within the FNCLCC ‘low/indeterminate’ risk category, which could assist identifying patients likely to benefit from peri-operative chemotherapy.

## Outstanding questions

The developments in prognostic transcriptomic signatures are very promising but there remain outstanding questions to be addressed. The vast majority of transcriptomic signatures in sarcoma have been developed from bulk measurements of tumour specimens. However, all the studies described to date have not systematically established if the performance of these prognostic signatures are influenced by the intratumoural heterogeneity inherent within STS^15,74^, for instance by evaluation of distinct heterogeneous tumour regions with spatial transcriptomics. Furthermore, there is a diversity of distinct cell types in the tumour microenvironment (e.g., immune cells, fibroblasts and endothelial cells) which can be readily assessed by histopathology review but is lost in bulk transcriptomic data. Emerging data from single cell RNA sequencing has started to shed light on the intratumoural heterogeneity inherent in some subtypes^75^, but the cost of deploying this technology in routine clinical setting is prohibitive. Alternatively, deconvolution methodologies may be used to establish distinct immune cell types from bulk transcriptomic data^76^. Given that these transcriptomic signatures have been established and validated in bulk measurements, whether the use of single cell or deconvolution analysis will lead to a loss in prognostic value remains an open question. From a biological perspective, it is also currently unknown if transcriptomic subgroups are retained during disease progression or treatment. For instance, it is unclear if CINSARC subgroups remain stable in patient-matched primary, locally relapsed and metastatic tumours and if the subgroup classification alters in response to therapy. This stability of subgroup has profound implications for implementation in clinical practice as it determines which point in the patient journey the prognostic assay should be applied. Finally, interlaboratory benchmarking for reliability and reproducibility of these complex transcriptomic assays needs to be undertaken across multiple laboratories prior to use as routine clinical assays.

## Future perspectives and conclusions

Use of transcriptomic biomarkers in sarcomas provides an exciting opportunity to improve the limited treatment options and dismal prognosis for patients with advanced metastatic STS^6^. Personalised medicine may be achievable, with allocation of therapy based on prognostic transcriptomic biomarker expression. Further prospective validation of CINSARC, GGI, and hypoxia gene signatures is required to advance these signatures for routine use. In order to improve STS prognostication, histology-specific and site-specific nomograms combining clinical and pathological tumour and patient characteristics, have been used since 2002^77,78^. Combining existing sarcoma nomograms with transcriptomic signatures may further improve accuracy of risk assessment^79^. Additionally, integration of gene expression analysis with other Omic profiling methodologies, such as proteomics and DNA methylation analysis, is also likely to improve prognostication with new biomarker-directed treatment opportunities^80–83^.

Beyond their use in the peri-operative setting, transcriptomic signatures may have utility in the context of next generation therapies. There are several biomarkers for targeted therapies such as PD-L1 expression for immune checkpoint inhibitors and PARP-1 levels for PARP inhibitors^84,85^. Integration of these biomarkers with transcriptomic signatures may be of prognostic benefit. Bertucci et al. assessed expression of PD-L1 and PARP-1 in separate retrospective series of genomically-complex STS, and found that tumours with high PD-L1 or PARP-1 expression had worse MFS^86,87^. These studies found that expression of these biomarkers could enhance prognostication with transcriptomic signatures. For example, PARP-1 expression complemented the prognostic value of CINSARC, with a 5-year MFS rate of 77% in C1/PARP1-low tumours compared to 43% in C2/PARP1-high cohort (*p* = 2.55 × 10^−11^). Through identifying high-risk patients, these integrated biomarkers could select patients who would benefit from targeted therapy and more aggressive treatment strategies.

A better understanding of orthogonal molecular features (such as copy number alterations and DNA methylation status) as well as the regulatory mechanisms driving the observed transcriptional signatures will be key to delineating sarcomagenesis and identifying potential therapeutic targets. Lesluyes and Chibon recently applied the CINSARC signature to the multi-Omics dataset from The Cancer Genome Atlas sarcoma cohort^23^. Focusing on the sarcomas with complex genetics, they showed that copy number alterations were significantly increased in C2 compared with the C1 group (*p* = 2.33 × 10^−2^), as was whole genome-doubling (*p* = 1.82 × 10^−2^) and higher ploidies (*p* = 3.94 × 10^−3^)^88^. Low whole-genome DNA methylation was associated with more aggressive C2 tumours. However, DNA methylation was not observed in CINSARC promoter regions which is indicative that this is not a direct regulator of CINSARC expression. Further, most miRNAs (67%) overexpressed in the C2 group were known oncomirs. Understanding the regulation, epigenetic or otherwise, of CINSARC gene expression is an exciting avenue of future translational research.

Much can be learned from progress in other tumour types where precision medicine is widely used in clinical practice^89^. In order to be approved for clinical use, gene expression signatures need to undergo a complex series of steps to demonstrate the required LoE in prospective clinical trials. Promising early steps have been undertaken with CINSARC, and we anticipate that wide adoption of transcriptomic signatures in sarcoma clinical practice will require international collaboration for robust prospective validation of their prognostic and clinical utility.
